# A call for strategy on eating disorders: the need for a comprehensive eating disorder strategy in England and specific guidance for the remote delivery of eating disorder services

**DOI:** 10.1186/s40337-025-01224-y

**Published:** 2025-03-27

**Authors:** Richard Brown, Claire Murphy-Morgan, James Downs, Dawn Branley-Bell

**Affiliations:** 1 Department of Psychology, Northumbria University, Newcastle upon Tyne, UK; 2 Peer Researcher and Expert by Experience, Cardiff, UK

**Keywords:** Eating disorders, Public health, Healthcare, Policy, NHS, Remote healthcare, Telehealth

## Abstract

There are growing calls for a comprehensive, evidence-based national eating disorder (ED) strategy for England. This is due to the rising prevalence of EDs, the lack of national guidance around different ED presentations (and potentially varying treatment needs), ad hoc data collection, and inconsistencies in both care and evaluation of service provision quality. Furthermore, the shift towards remote delivery of care during the COVID-19 pandemic underscores the need for government strategy to include specific guidance on remote delivery of ED services. The increased use of remote healthcare presents an opportunity to mitigate regional disparities in the provision of care. However, there are distinct challenges when delivering ED services remotely. In this position paper, we firstly highlight the growing need for a comprehensive national ED strategy to combat the rising prevalence and harm of EDs. Secondly, we specify the importance of ensuring that future governmental strategy incorporates evidence-based guidelines specific to remote delivery of ED services. This is crucial for promoting consistent provision of ED care. We set out the lack of comprehensive national data, and the need for further research into remote service delivery.

## Introduction

Eating disorders (EDs) are a major public health concern for the United Kingdom (UK), with an estimated 1.25 million people in the UK having an ED [1]. The total financial cost of EDs to the English economy in 2020 was estimated at £8 billion (including National Healthcare Service [NHS] expenditure, carer costs, personal finance costs, and productivity loss) [2]. The Mental Health of Children and Young People (MHCYP) survey estimates that 12.5% of 17 to 19-year-olds in England reported having an ED in 2023, compared with 0.8% in 2017 [3]. During this time, rates amongst this age group rose from 1.6 to 20.8% in women and from < 0.1 to 5.1% in men [3]. These rates are likely to be a significant underestimation due to most individuals who experience EDs never seeking help [4–6].

The NHS in the UK serves as the overarching term for the health systems of England, Scotland, Wales, and Northern Ireland. Since the transfer of powers to each of the devolved nations in 1999, health matters have largely fallen under their separate jurisdictions [7]. This paper calls for the Government to set out a comprehensive strategy for tackling EDs in England. Here we describe current services and levels of performance, pathways to improvement, and the Government’s approach to public health. We also echo recent calls [8, 9] for the Government to provide a national strategy to combat the rising prevalence of EDs in England. In 2019, the Public Administration and Constitutional Affairs Committee reported that, “*the lack of precise information on the prevalence of eating disorders is shocking*” and that this, “*limits the ability of NHS commissioners to gauge what services need to be provided and encourages them to devote resources to better recorded diseases and conditions*” [10]. There have been calls for increased collection and availability of data by Members of Parliament (MPs) from across the political spectrum [11] to facilitate research and provide a better understanding of experiences of EDs across the country.

EDs increase the risk of premature death, with anorexia nervosa having a higher mortality rate than any other mental health disorder [1, 12]. Early diagnosis and treatment are crucial [13]. However, several barriers to support have been highlighted, such as the level of stigma still associated with EDs [14] and a widespread lack of understanding amongst primary healthcare professionals (including around varying types of ED presentations) [15, 16]. Guidance from the Royal Society of Psychiatrists (2022) sets out that BMI (Body Mass Index) should not be the sole indicator in assessing the need for ED treatment, yet there remain many instances whereby individuals report that their referral for treatment appears to be based on BMI [8, 17]. Even for diagnosed patients who can access NHS services, delivery of care, including the provision of care pathways for long-standing and severe illness, is varied [18, 19]. There is also a worrying trend towards identifying some patients as ‘untreatable’ including an emergent controversial discussion regarding whether palliative care is an appropriate treatment response in some ED cases [20–22]. The number of individuals without a formal ED diagnosis due to not being able to access NHS services is also growing [15, 23–25]; often attributed to a lack of local services, or services being stretched to capacity [18, 24, 26]. Additional barriers to treatment have also been experienced by marginalised or underserved communities. For example, there is evidence of insufficient support for minority ethnic individuals due to delayed recognition of EDs within these communities by service providers, and limited service capacity and/or understanding to address culturally specific factors critical for diagnosis and recovery [27, 28]. Young people with minority sexual orientations and/or gender identities can also face barriers in accessing care, including a lack of discrimination-informed approaches [29, 30]. Furthermore, men with EDs are also often underserved by ED services due to persistent misconceptions that EDs ‘*only affect women*’ [31, 32]. These barriers to support illustrate the need for more equitable and person-focused interventions [2].

### Government strategy for public health

In 2017, the Parliamentary and Health Service Ombudsman (PHSO) published their damning assessment on ‘*How NHS eating disorder services are failing patients’*; making several recommendations for improving care [33]. However, in 2023, the PHSO stated that the Government had made “*little progress*” in implementing the recommendations and that, since the beginning of April 2019, the PHSO had received 234 complaints about ED services [34].

The 2019 NHS Long Term Plan [35], declares a commitment to improving treatment for mental health, including EDs, and a ringfenced local investment fund worth “*at least £2.3bn a year by 2023/24*” [36]. However, the plan was created before the COVID-19 pandemic, which subsequently changed the landscape of healthcare delivery. In August 2023, the previous Government’s interim report on its Major Conditions Strategy stated that mental ill health would be prioritised as part of the framework to target six groups of major health conditions (cancers, chronic respiratory disease, cardiovascular disease, dementia, musculoskeletal disorders, and mental ill health) [37]. EDs were featured in multiple health policies drafted by the previous Conservative Government, including the suicide prevention strategy for England, in 2023 [38], which states that one-quarter to one-third of people diagnosed with anorexia nervosa or bulimia nervosa have attempted suicide, and that the Government “*intends to explore opportunities to improve the quality of care for patients with these diagnoses”.* The 2022 Women’s Health Strategy for England [39] also declared a commitment to developing a deeper understanding of the causes of mental ill health, including conditions with higher rates of prevalence in women, such as EDs. No equivalent strategy currently exists for men’s health, despite growing calls from politicians and campaigners [39, 40] and research indicating that around 1-in-4 of those living with an ED are male [41, 42].

Despite reference to the importance of addressing EDs, the previous Government did not present a clear strategy for ED services in England. Such strategies have been published in other countries. For example, the National Eating Disorders Collaboration (NEDC), funded by the Australian Government, published the National Eating Disorders Strategy 2023–2033 aiming to provide a nationally consistent, evidence-based system of care and highlight the importance of remote ED services [43]. Similarly, the National Public Health Service for Wales set out its ED Framework with the Welsh Government conducting a wide-ranging service review in 2018 [44, 45]. More recently, in November 2024, the Scottish Government published a National ED Specification that “*outlines a national baseline of eating disorder service provision for the delivery of person-centred*,* safe*,* and effective care*” [46]. The specification was developed to complement broader mental health policies and was drafted in response to a national review of ED services and extensive public consultation [47].

### A national eating disorder strategy

In 2022, the Health and Social Care Committee (HSCC) recommended *“that the Government develops a national eating disorder strategy that aims to understand the causal mechanisms that lead to the development of eating disorders and earmarks adequate funding to bolster existing services as well as to increase investment in research”* [48]. Further calls from academics have encouraged the Government to facilitate increased research and innovation targeting improvements to ED services [49–51]. In 2023, a mounting demand emerged from activists [52] and politicians [9, 53] for the Government to formulate a national strategy for tackling EDs. The HSCC argued that a full ED strategy would help to eliminate regional inequities in care provision [48]. The previous Conservative Government responded to the Committee’s report by stating, “*as we expect eating disorders will be considered as part of this work [the Major Conditions Strategy]*,* the Government does not intend to publish a separate national eating disorder strategy*” [54]. However, incorporating ED policy into a general approach is likely to overlook unique intricacies involved in successfully delivering ED services. EDs bring very nuanced challenges which may be missed by broad stroke “*one size fits all*” approaches [31, 55, 56]. Cancer Research UK’s Chief Executive, Michelle Mitchell, raised concerns about the previous Government’s approach, stating that it has “*opted to publish a ‘catch-all’ major conditions strategy*” and that this approach may “*further dilute*” government commitments to addressing specific health conditions [57]. In July 2024, the King’s Speech, which sets the legislative agenda for the following parliamentary session, highlighted that the new Labour Government are committed to modernising the Mental Health Act, reducing NHS waiting times and giving mental health the same attention as physical health [58]. However, the new Government has yet to make any specific statements regarding their plans for ED services or strategies.

We acknowledge that implementing a dedicated public health strategy to improve ED services, as seen in other countries, would require extensive consultation processes and significant government expenditure. Furthermore, efforts to address EDs in England have been made through policies on women’s health [39], online safety [59], and primarily mental health [3, 37, 60]. However, EDs are distinct from many other mental health conditions, not only in having the highest mortality rate [1, 12], but in that they have more frequent involvement of medical comorbidities [61]. A lack of research has led to critical gaps in our understanding of the multiple effects of EDs on physical changes to the cardiovascular system, bone density, and gastrointestinal complications [61]. The interrelationship between mental and physical health in the manifestation and effects of EDs can also complicate access to treatment [20]. Physical and behavioural metrics (such as weight, BMI, or frequency of binging/purging episodes) are often used to assess need for treatment. However, this can fall short of considering emotional and psychological impacts, leaving little space for the patient to discuss this with a clinician. EDs may also be uniquely influenced by harmful online content, particularly with frequent online discourse around weight, diet, fitness and body shape [62–64] and a rise in augmented and artificial representations of body image [62–64]. In 2024, NHS England carried out a public consultation on proposed changes to the Specialised Adult Eating Disorder Inpatient Service [65]. In anticipation of the transition of services to the NHS Integrated Care Boards model (due 2025), the consultation proposes a more multidisciplinary and holistic approach to care. Whilst the proposed changes go some way to providing greater consistency across England, proposals are in-patient focused. A similar approach is lacking when it comes to supporting individuals without a diagnosis– a growing population that would benefit from early intervention support [15, 23–25]. Hence the need for a broader ED strategy going forward.

### The benefits of precise strategic planning

Clear and distinct strategies for specific health issues can help governments to develop long-term approaches to tackling societal problems and can engage the public voice in long-term planning to address complex issues [66]. General health strategies may fail to go beyond generic measures of success (e.g., number of patients in treatment, and patient waiting times). The use of alternative metrics, in dedicated policies, can better assess the long-term success of health policies (e.g., patient experience, treatment effectiveness) [67, 68]. This can support the assessment of effective provision and help address challenges when it comes to embedding services at primary care level (e.g., roll out costs, scalability, and implementation). Data can provide insights into how to create and embed specialised services in voluntary or lower tier services and promote a joined-up approach between primary care and additional providers (whilst also improving understanding of what good treatment looks like).

## Specific guidance needed for remote delivery of eating disorder services

A national ED strategy for England must also include guidance around *remote* delivery of services. Here we explain why this change to the healthcare landscape leads to nuanced concerns, the problem of inconsistent service delivery, and how this relates to health inequities.

### The rise in the remote delivery of health services

The COVID-19 pandemic rapidly accelerated the adoption of remote healthcare [69–71], far outpacing the timeline anticipated in the NHS Long Term Plan [72]. While the NHS remains committed to boosting investment in ED services, neither the NHS Long Term Plan [36], the more recent Long Term Workforce Plan [73], nor the Mental Health Implementation Plan [60] specifically address remote delivery. Instead, guidance relevant to remote care is primarily found within broader Government and NHS commitments to improving mental health treatment and advancing the digital transformation of the NHS [74].

Existing evidence-based protocols for delivering ED services have focussed on face-to-face treatment [75]. As a result, those offering ED services had to quickly adjust their methods of delivery with little formal guidance, leading to disparities in access and quality of care [76]. While face-to-face delivery has resumed, levels of remote delivery remain significantly higher than pre-pandemic. In 2024, 58.04% of contacts with mental health services by children and young people were face-to-face (*n* = 3,493,814 / 6,019,666 total attended contacts), dropping from 75.82% in 2018 (*n* = 2,686,797 / 3,543,652 total attended contacts) [77]. Remote healthcare is expected to remain, as service providers and users have expressed continued need for online and hybrid support [24, 78]. Comprehensive data on the percentage of ED services in England delivered remotely are currently unavailable. Data is crucial for assessing both current and longer-term accessibility, particularly for underserved communities, and ensuring consistency of care nationwide. It plays a key role in addressing regional disparities.

The NHS’ 2023 Digital Maturity Assessment programme aims for a “*consistent and cost-effective approach to remote consultations*,* monitoring and care services*” that promote “*patient choice and sustainability*”. However, the HSCC have rated the Government’s progress in implementing its commitments to the digitisation of the NHS as “*inadequate*”, highlighting failures to fulfil commitments to roll out integrated health and care records, develop mechanisms to de-identify data on collection from GP practices, and create a national digital workforce strategy [79]. Furthermore, the Committee of Public Accounts’ report on progress in improving NHS mental health services raised several concerns, including highlighting the “*patchy implementation of clinical guidance across local areas for people with eating disorders*” [80].

### Specific concerns about the remote delivery of eating disorder services

Remote support services can help to address some barriers to seeking help including stigma, cost, limited mobility, and barriers to access (including those with caring responsibilities, and in rural or underserved areas) [81–83]. Since most individuals experiencing EDs never receive formal help [16, 23–25], remote care can help to address this critical issue. Remote healthcare presents several common challenges, such as information loss, communication problems, difficulty building rapport, privacy concerns, and limited access to services [76, 84]. However, there are also more nuanced challenges when it comes to remote ED services [24, 84, 85] including negative impacts of remote platform functionality on body image, limited ability to participate in group or peer support sessions (e.g., shared mealtimes), challenges in accurate weight and symptom monitoring [71, 75, 84], presentation of exacerbated symptoms due to increased isolation, and concerns around increased ability to mask ED symptoms [24, 70].

Video conferencing platforms are one of the most widely used forms of remote ED support, but they present several challenges. For example, these platforms typically have self-view (where the individual can see their own video on the screen during the call) as the default option. This can lead to increased self-evaluation [71, 76, 86, 87], particularly concerning for people with EDs [71, 88]. Some patients may experience feelings of shame or discomfort in response to seeing their image onscreen [84], potentially triggering ED thoughts or behaviours [24, 76, 86]. Whilst cameras can be turned off, this can in some cases facilitate individuals concealing or masking the severity of their symptoms, leading to concerns around effective monitoring. This is a particular concern as secrecy and isolation can often allow an ED presentation to take hold [24, 75, 86]. Unbeknown to many service users and service providers, many popular platforms offer the option to switch off ‘self-view’. This can be done without disabling the whole camera (i.e., the recipient can still see the individual’s video, but the individual is not shown their self-view). This allows for a more natural face-to-face interaction (where we are not able to see ourselves when talking to another person). This can increase comfort and reduce risk of critical self-evaluation but still allow the service provider to see the individual. Research has called for practical guidance and education for service providers and users, including how to turn-off self-view videos [89] and researchers have developed open-access, co-designed tools aiming to support this (e.g., The ‘ConnectED on the Journey’ toolkit, www.rhedc.uk/toolkit). It is important to note here that careful consideration is needed to identify when it may be appropriate to request that service users do not disable self-view [71]. Although self-view can be problematic, it may also offer opportunities for remote treatment. For example, exposure therapy is possible using video conferencing platforms. Exposure therapy is often delivered in the context of Cognitive Behavioural Therapy (CBT) and includes techniques such as mirror therapy, where an individual is exposed to looking at themselves in a mirror (or in this instance via self-view) in a supported environment with a healthcare professional. Over time, the treatment aims to reduce associated fear, anxiety and/or distress through repeated, supported exposure. Mirror therapy is possible via remote platforms, although this can be challenging [75]

Physical monitoring by service providers can remain a challenge, even when cameras are switched on; for example body appearance can be affected by lighting, camera angles, or clothing. In response to these challenges, some individuals were requested to ‘self-weigh’ during the pandemic lockdown periods and asked to report their weight back to their service provider [26]. Self-weighing is not recommended and can cause heightened levels of stress and anxiety [90–93]. Self-weighing is very different to being weighed at a face-to-face appointment where a medical professional is present to offer support, and where blind weighing can be used. Many individuals with EDs choose, and are often advised, not to keep scales in their household as self-weighing can lead to the exacerbation of ED symptoms [76]. These nuanced challenges highlight where specific guidance would be beneficial.

It is also vital to acknowledge and adequately address the digital divide when offering remote service provision. The digital divide refers to the gap between people who can readily access digital technologies, including the internet, and those who cannot [94]. This may be due to limited access to digital technology or for other reasons such as low digital literacy skills. The digital divide is heavily impacted by socioeconomic status [94]. According to the Lloyds Bank UK Consumer Digital Index 2021 survey [95], over one-third of UK benefit claimants have very low digital engagement, and millions of people across the UK struggle to engage with remote health support services. Inequalities can be reinforced or exacerbated if remote services are more easily accessed, adopted or adhered to by already advantaged societal groups [96–99]. Therefore, guidance on remote healthcare delivery, both within and outside of ED specific guidance, must ensure that it acknowledges the digital divide and proposes a clear strategy to address health inequity challenges.

### The potential of remote support in addressing inconsistencies in service delivery

Disparities exist in both the provision and quality of ED services across different regions of England, creating a ‘*postcode lottery*’ [100, 101]. More affluent areas, such as in London and the surrounding counties, typically have greater funds available for healthcare [102, 103]. As a result, individuals with lower socioeconomic status are less likely to be diagnosed and treated for an ED [103, 104]. Effective remote delivery could help to address regional disparities in provision by broadening the reach and accessibility of services. However, without a clearly defined national standard, we argue that further implementation could exacerbate, not reduce, health inequalities [19]. There must also be a consistent approach to hybrid care (integrated in-person and remote care) as research indicates the importance of remote care as a compliment to, not a replacement for, in-person services [78]. In-person multidisciplinary teams (e.g., clinicians, dieticians, therapists) have a role to play in ensuring a holistic package of support.

Third sector organisations are often a lifeline for individuals looking for support who may not be able to access NHS services (whether due to regional disparities in access, increased wait times, perceptions of “*not being ill enough*” and/or not meeting pre-defined criteria for access). Charities report being overwhelmed by demand during, and since, the pandemic [78, 105]. Diagnosis and clinical treatment are not routinely within the remit of third sector services, yet there is evidence that they are now being called upon to provide remote support for individuals who, pre-pandemic, would have been deemed too unwell for their services [78]. An example of how the charity sector has stepped up is First Steps ED’s ‘Waiting Well’ Service [106], a monthly online support group developed to help offer support whilst waiting for NHS services. This is an example of the growing need for both statutory health and not-for-profit organisations to collaborate in meeting growing demand [107]. The challenge with this approach is ensuring that not-for-profit organisations are adequately funded for a sustained period to be able to develop and provide such services, and that individuals who require more specialist support are provided quicker access to appropriate services. A national ED strategy could help to standardise collaborative working between NHS and voluntary sector partners and go some way towards alleviating the ‘*postcode lottery*’ of provision.

## Conclusions

In this paper we have supported calls made by activists [52], politicians [8, 9, 53], and the Health and Social Care Committee [48] for the Government to implement a national ED strategy for England - aimed at tackling the rise in prevalence of EDs and disparities in the provision of care. We have also echoed calls for increased collection and availability of data regarding ED services. Improved data would faciliate monitoring of regional differences in the type and quality of service provided, as well as helping to capture the full extent of ED prevalence in England. The shift towards remote delivery of care has underscored the need for government strategy to include specific guidance on remote ED services. We have specified the importance of evidence-based guidelines specific to remote delivery of ED services and continue to argue that these guidelines should be incorporated into a national ED strategy. In sum, a dedicated and clearly defined national strategy is needed to tackle EDs in England and should aim to ensure equitable and effective delivery of remote services. People with lived experience of EDs, their supporters and carers, should be at the heart of co-designing this strategy.

## Data Availability

No datasets were generated or analysed during the current study.
